# Nest use dynamics of an undisturbed population of bald eagles

**DOI:** 10.1002/ece3.4259

**Published:** 2018-06-27

**Authors:** Tammy L. Wilson, Joshua H. Schmidt, Buck A. Mangipane, Rebecca Kolstrom, Krista K. Bartz

**Affiliations:** ^1^ Southwest Alaska Network National Park Service Anchorage Alaska; ^2^ Department of Natural Resource Management South Dakota State University Brookings South Dakota; ^3^ Central Alaska Network National Park Service Fairbanks Alaska; ^4^ Lake Clark National Park and Preserve National Park Service Port Alsworth Alaska

**Keywords:** bald eagle, *Haliaeetus leucocephalus*, hierarchical Bayesian model, long‐term monitoring, multistate model, population dynamics

## Abstract

Management or conservation targets based on demographic rates should be evaluated within the context of expected population dynamics of the species of interest. Wild populations can experience stable, cyclical, or complex dynamics, therefore undisturbed populations can provide background needed to evaluate programmatic success. Many raptor species have recovered from large declines caused by environmental contaminants, making them strong candidates for ongoing efforts to understand population dynamics and ecosystem processes in response to human‐caused stressors. Dynamic multistate occupancy models are a useful tool for analyzing species dynamics because they leverage the autocorrelation inherent in long‐term monitoring datasets to obtain useful information about the dynamic properties of population or reproductive states. We analyzed a 23‐year bald eagle monitoring dataset in a dynamic multistate occupancy modeling framework to assess long‐term nest occupancy and reproduction in Lake Clark National Park and Preserve, Alaska. We also used a hierarchical generalized linear model to understand changes in nest productivity in relation to environmental factors. Nests were most likely to remain in the same nesting state between years. Most notably, successful nests were likely to remain in use (either occupied or successful) and had a very low probability of transitioning to an unoccupied state in the following year. There was no apparent trend in the proportion of nests used by eagles through time, and the probability that nests transitioned into or out of the successful state was not influenced by temperature or salmon availability. Productivity was constant over the course of the study, although warm April minimum temperatures were associated with increased chick production. Overall our results demonstrate the expected nesting dynamics of a healthy bald eagle population that is largely free of human disturbance and can be used as a baseline for the expected dynamics for recovering bald eagle populations in the contiguous 48 states.

## 1 INTRODUCTION

Monitoring is a necessary part of any management or conservation program; providing the means to evaluate goals while also accounting for long‐term system dynamics and interannual variation (Stem, Margoluis, Salafsky, & Brown, 2005). Species persistence is a popular target of both conservation and management; however, it can be difficult to evaluate programmatic success because populations can be stable, cyclical, or chaotic, based on both intrinsic and extrinsic factors that often interact (Bjørnstad & Grenfell, 2001). Gains in abundance and fecundity can be expected to decline as populations experience density dependence (e.g., Ferrer & Donazar, 1996). However, demographic metrics of cyclical populations will also vary with the period of the cycle (e.g., Krebs, Boonstra, Boutin, & Sinclair, 2001; Schmidt, McIntyre, Roland, MacCluskie, & Flamme, 2018). Further, demographic rates can depend on the underlying age‐structure of the population, causing complex dynamics, including occasional sharp declines (e.g., Coulson et al., 2001). These dynamics suggest that it is unrealistic to expect that recovering populations produce continued increases in abundance or maintain high fecundity as populations approach recovery. Therefore, data about population dynamics from undisturbed populations (i.e., not subjected to excessive stressors) can provide context that can help to evaluate the progress of conservation or management programs tasked with recovering or maintaining species abundances.

**Figure 1:**
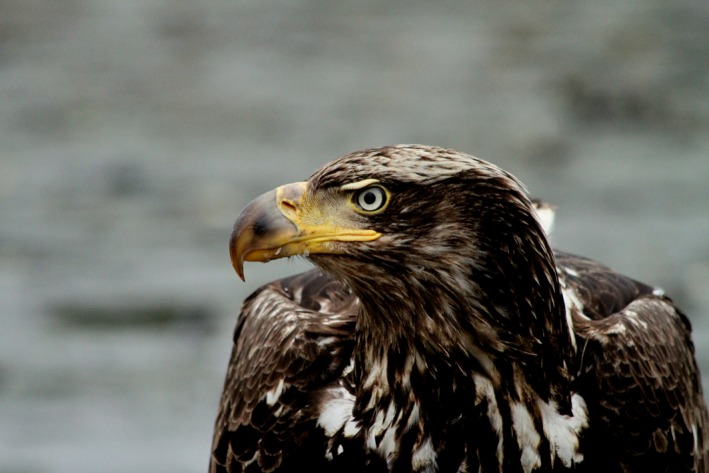
Immature bald eagle in Alaska, USA. Photo by Chris Sergeant, NPS

Many raptor populations have been the subject of extensive monitoring efforts for decades due in part to a history of population declines (Kirk & Hyslop, 1998; Snyder, Snyder, Lincer, & Reynolds, 1973), their sensitivity to pollution (Grasman, Scanlon, & Fox, 1998), and their responses to anthropogenic disturbance (Steidl & Anthony, 2000). In particular, the bald eagle (*Haliaeetus leucocephalus*; Figure 1) experienced substantial population declines due to human‐caused disturbance. Although illegal shooting certainly impacted populations prior to the enactment of the Bald Eagle Protection Act of 1940 (16 U.S.C. 668‐668c), reductions in nesting success and productivity due to environmental contaminants (Dykstra et al., 2001; Grasman et al., 1998; Grier, 1982) were largely responsible for bald eagles being listed under the Endangered Species Act in most of the conterminous United States (43 FR 6233, 1978). Bald eagle populations have recovered in the wake of banning DDT, and the bird was removed from the endangered species list in 2007 (72 FR 37346).

Although considered to be recovered, some eagle populations are still at risk from a suite of environmental contaminants (Bowerman et al., 2003; Venier, Wierda, Bowerman, & Hites, 2010), and high toxin loads have been recorded in Alaska (Anthony, Miles, Ricca, & Estes, 2007). Further, direct human disturbance can cause changes in spatial use patterns and activity budgets of bald eagles, which can negatively affect reproduction (Cain, 2008; Fraser & Anthony, 2008; Steidl & Anthony, 2000) and nest site fidelity (Fraser & Anthony, 2008). In some areas, bald eagles are directly monitored as bioindicators of toxic chemicals (Route, Bowerman, & Kozie, 2009). It is more common, however, that local populations are monitored to generate information on population dynamics, trend, and habitat requirements of the recovering population as a whole (e.g., Smith, Hess, & Afton, 2016; Watts, Therres, & Byrd, 2008; Wilson, Schmidt, Thompson, & Phillips, 2014).

Bald eagle nesting, nest success, and chick production are dependent on various factors including food availability and weather conditions (Gende & Willson, 1997; Hansen, 1987). These factors can also interact to influence hatching dates and reproductive rates, as observed in golden eagles (*Aquila chrysaetos*) by Steenhof, Kochert, and McDonald (1997). Variation in observed nesting, success, and productivity caused by these factors can inhibit our ability to detect directional changes in monitored metrics that would indicate impacts to a population. Therefore understanding the causes of interannual variation and dynamic properties of nest use is important for effective species conservation (Dale & Beyeler, 2001).

Bald eagle pairs mate for life and exhibit high degrees of fidelity to nest sites (Jenkins & Jackman, 1993; Stalmaster, 1987). Nest reuse may provide reproductive benefits, such as a higher probability of breeding success or more fledglings in pairs of birds reusing nests (Jiménez‐Franco, Martínez, & Calvo, 2014). However, nests that have not been reused for many years provide little value (Watts, 2015). The long‐term dynamics of nest use by eagles is not well understood, and obtaining a holistic picture of nest use will improve inference from long‐term studies. Dynamic multistate occupancy models are a powerful tool for understanding how breeding status changes at sites through time (MacKenzie, Nichols, Seamans, & Gutierrez, 2009), and such models have been successfully used to monitor occupancy and breeding dynamics in a variety of species (Kroll, Jones, Stringer, & Meekins, 2016; Martin et al., 2009; Schmidt, Flamme, & Walker, 2014). These models use observations of distinct reproductive states, which are usually much easier to obtain than detailed reproductive rate data (i.e., fecundity), and leverage autocorrelation that is inherent in monitoring data to estimate state transition probabilities and identify factors influencing them (Kroll et al., 2016; MacKenzie et al., 2009).

Using a dynamic multistate occupancy modeling approach, we analyzed a 23‐year bald eagle nest monitoring dataset from Lake Clark National Park and Preserve (LACL). The dataset provided an opportunity to assess the temporal properties of bald eagle nest use and chick production in a population subject to minimal human disturbance. Our specific objectives were to: (1) assess the occupancy dynamics of nesting bald eagles in LACL; (2) determine if interannual variation in occupancy dynamics was related to local environmental conditions or food availability; and (3) investigate whether population metrics showed evidence of a trend through time. We hypothesized that eagle nest occupancy dynamics and productivity would be affected by environmental and biological factors during nest initiation and incubation. We also expected that the dynamics of a population regulated by density‐dependent factors would be stable through time. Our results establish baseline expectations for a bald eagle population that is largely free from human interference during nesting. These metrics will be useful as targets for impacted and recovering populations elsewhere.

## 2 MATERIALS AND METHODS

### 2.1 Ethics statement

In Alaska, bald eagles are protected under the Bald and Golden Eagle Protection Act (1940), the Migratory Bird Treaty Act (1918), and the Lacey Act (1900). Eagles are not directly handled during monitoring, and flights are conducted in a way to minimize the disturbance to nesting eagles. Our methods closely follow the United States Fish and Wildlife Service's Post‐Delisting Monitoring Plan (U.S. Fish and Wildlife Service 2009) and are described in the accepted NPS protocol for monitoring bald eagles in the Southwest Network (Wilson, Weiss, Shepherd, Phillips, & Mangipane, 2017).

### 2.2 Study area

Lake Clark National Park and Preserve is located at the intersection of the Alaska and Aleutian Mountain ranges in southwestern Alaska. The climate is representative of the southern boreal forest, which consists of cold winters and cool, wet summers. The average minimum April temperature (when eagles begin to occupy nests) in the National Oceanic and Atmospheric Administration (NOAA) Bristol Bay climate division (Bieniek et al., 2012) was −20°C (1993–2015), increasing to a maximum of 15°C in July, the warmest part of the nesting period. Most precipitation fell as rain. During the study period, the average precipitation in May when eagles were incubating eggs was 58 mm (1993–2015). Bald eagles generally feed on waterfowl, marine birds, terrestrial mammals, and a variety of intertidal, and freshwater fishes (Knight, Randolph, Allen, Young, & Wigen, 1990), but the 5 species of Pacific salmon (*Oncorhynchus* spp.) are numerically dominant in eagle diets (Hansen, 1987). Bald eagles were found throughout the park, nesting in white spruce (*Picea glauca*) and cottonwood (*Populus tricocarpa*) trees along lake shores, streams, and coastlines; ground nests were found occasionally.

### 2.3 Data collection

**Figure 2:**
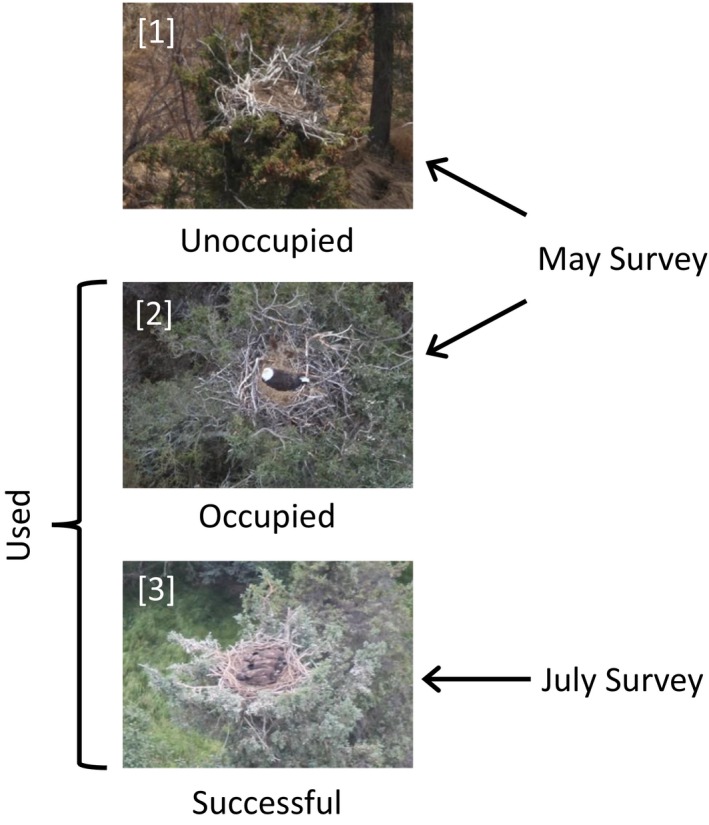
Diagram of the terms used to describe the occupancy states used to examine bald eagle nest dynamics for 23 years in Lake Clark National Park and Preserve, Alaska, USA

We conducted surveys of bald eagle nests twice annually from a small fixed‐wing aircraft (e.g., Piper Aviation super cub‐ PA18) from 1993 to 2015, except in 1998, 2002, and 2003 when weather or other logistical considerations prevented one or both surveys. During the first survey, we visited all known nests in early May to observe nest occupancy by eagles. During the second survey in late July or early August, we returned to nests where we observed nesting eagles during the first survey to assess nest success and count fledgling chicks. Based on these 2 surveys, we classified nests into three mutually exclusive occupancy states (Figure 2) using terminology similar to that developed in the multistate occupancy literature (MacKenzie et al., 2006, 2009). The three possible states for each observed nest were: (1) unoccupied (no evidence of nesting activity), (2) occupied (eggs or incubating adult observed during the first survey, no eaglets present during the second survey), and (3) successful (one or more live eaglets observed during the second sampling period). We considered a nest to have been used if it was observed in either the occupied or successful state. Our state definitions apply to individual nests because of the difficulty of defining territory boundaries in high‐density bald eagle populations in Alaska (Hodges, 1982).

New nests were added to the list of known nests opportunistically through time as they were discovered. We also conducted a systematic search for new nests in 2012 to ensure that large numbers of unknown nests were not present (<10 new nests added). Occasionally known nests were not observed; these nests were coded as not available (NA). Nests that had not been seen in 3 years were not searched for again, and the nest state was coded as not available (NA). These missing nests remained in the dataset, but contributed no information to the posterior distribution. The chick count data were analyzed separately as described below.

We obtained April minimum temperature and mean May precipitation data from the NOAA National Climatic Data Center (now called National Centers for Environmental Information‐ NCEI) monthly climate division data (http://www1.ncdc.noaa.gov/pub/data/cirs/climdiv accessed 10 September 2017). These covariates were chosen to represent periods in the nesting cycle during which eggs or small nestlings would be most vulnerable to temperature and precipitation extremes based on our reading of the raptor literature. We used the Bristol Bay climate division because a majority of Lake Clark is located within it, as are the nearest weather stations used to produce the climate summaries. We considered the number of migrating adult sockeye salmon (*Oncorhynchus nerka*) that escaped the marine fishery to spawn in freshwater (salmon escapement) to be a suitable measure of the amount of food available to bald eagles each year from 1992 to 2015 (http://www.adfg.alaska.gov/sf/FishCounts accessed April, 2016; Division of Commercial Fisheries 2015; Elison et al., 2015). We used the Kvichak counts because they were complete for the time period of interest, and covered the majority of nest locations in LACL. All continuous variables were centered and scaled to ensure that the intercept and effect sizes were more directly interpretable and to improve model convergence.

### 2.4 Data analysis

We analyzed the data using a dynamic multistate occupancy model (MacKenzie et al., 2009; Royle & Kery, 2007; Schmidt et al., 2014, 2018) that allowed nests to transition among occupancy states between years as a function of both temporal and spatial covariates. We assumed that the occupancy state of each nest was observed without error and adopted a deterministic model. This assumption was required because only occupied nests were revisited to evaluate success, and we therefore lacked the replicate data to formally model state uncertainty. Violation of this assumption would result in additional heterogeneity in transition probabilities and negative bias in the proportion of nests assigned to higher occupancy states (i.e., occupied or successful). However, this bias was expected to remain consistent through time, allowing us to generate unbiased estimates of change through time. Extra heterogeneity would also be expected to produce more conservative estimates of covariate effects.

Annual state membership, *y*_*it*_, for each nest, *i*, in each year, *t*, were constrained to sum to one:

$$y_{\mathrm{it}}∼\text{categorical}\;\left(\psi_{t}^{\left[s\right]}\right)$$

where membership in each of the three occupancy states (*s*) is mutually exclusive (i.e., nests can only exist in one state), and the vector ($\psi_{t}^{\left[s\right]}$) describes a complete row in a state and transition matrix (1 = unoccupied, 2 = occupied, 3 = successful). Recognizing that for a nest to be successful it must also be occupied, the probability of nest membership in each state $\psi_{t}^{\left[s\right]}$ was described by:

$$\psi_{t}^{\left[1\right]}=1-\left(\phi^{\left[2\right]}+\gamma^{\left[1][2\right]}+\gamma^{\left[3][2\right]}\right),$$

$$\psi_{t}^{\left[2\right]}=\left(\phi^{\left[2\right]}+\gamma^{\left[1][2\right]}+\gamma^{\left[3][2\right]}\right)*\left(1-\left(\phi^{\left[3\right]}+\gamma^{\left[1][3\right]}+\gamma^{\left[2][3\right]}\right)\right),$$

$$\psi_{t}^{\left[3\right]}=\left(\phi^{\left[2\right]}+\gamma^{\left[1][2\right]}+\gamma^{\left[3][2\right]}\right)*\left(\phi^{\left[3\right]}+\gamma^{\left[1][3\right]}+\gamma^{\left[2][3\right]}\right),$$

Where $\phi^{\left[s\right]}$ represented the probability of remaining in the same state, and $\gamma^{\left[s_{t-1}\right]\left[s_{t}\right]}$ was the probability of transitioning from one state to another in the next year. The cell probabilities for the full state and transition matrix are presented in Table 1. Transitions were fixed at the observed state for the first year (1993).

**Table 1:** Cell probabilities for the state and transition matrix of bald eagle nest occupancy states in Lake Clark National Park and Preserve, Alaska, USA. The rows indicate the state at year *t*−1 and the columns indicate the state at year *t*. The cells are the probabilities of either staying in the same state or transitioning to a new one. Each row denotes a true probability and sums to 1

	Unoccupied [s_t‐1_][1]	Occupied [s_t‐1_][2]	Successful [s_t‐1_][3]
Unoccupied [1] [s_t_]	1−(γ^[1][2]^ * (1−γ^[1][3]^) + γ^[1][3]^ * (1−γ^[1][2]^))	γ^[1][2]^ * (1−γ^[1][3]^)	γ^[1][3]^ * (1−γ^[1][2]^)
Occupied [2] [s_t_]	(1−ϕ^[2]^)* (1−γ^[2][3]^)	ϕ^[2]^	(1−ϕ^[2]^) * γ^[2][3]^
Successful [3] [s_t_]	(1−ϕ^[3]^) * (1−γ^[3][2]^)	(1−ϕ^[3]^) * γ^[3][2]^	ϕ^[3]^

We constrained all $\phi^{\left[s\right]}$ and $\gamma^{\left[s_{t-1}\right]\left[s_{t}\right]}$ to values between 0 and 1, and presented a logit‐linear model with parameters **α**_s_ and **β**_*s*,_ as follows:

$$\text{logit}\;\phi^{\left[s\right]}=\alpha_{s}+\beta_{s}*X_{\mathrm{it}}$$

$$\text{logit}\;\gamma^{\left[s_{t-1}\right]\left[s_{t}\right]}=\alpha_{s}+\beta_{s}*X_{\mathrm{it}}$$

where **α**_s_ describes a vector of the mean value of the state (2) or transition parameters (4), **β**_s_ is a vector of slope parameters, and **X**_it_ is a vector of time‐ and nest‐specific covariates. We used compact, normal priors for all regression parameters: **α**_*s*_ ~ Norm (0, 2.5) and  **β**_*s*_ ~ Norm (0, 2.5). We also derived estimates of the probability of nests being used by eagles, ($\psi_{t}^{\left[2\right]}+\psi_{t}^{\left[3\right]}$), and the probability of nest success conditional on use, $\left(\frac{\psi_{t}^{\left[3\right]}}{\psi_{t}^{\left[2\right]}+\psi_{t}^{\left[3\right]}}\right)$, to facilitate comparisons with past work.

To further evaluate productivity through time, we estimated the number of chicks produced in the average successful nest each year, $\lambda_{t}$. The general model can be written as:

$$\log\left(\lambda_{t}\right)=\alpha_{t}+\beta *X_{\mathrm{it}}$$

where the intercept is modeled as a random effect

$$\alpha_{t}∼\text{Norm}\;\left(\mu ,\sigma^{2}\right)$$

with mean μ and variance σ^2^. The expected number of chicks produced in successful nests was expected to vary as a function of the weather, and salmon escapement covariates, **X**_it_. We specified vague priors for all regression coefficients and hyperparameters : **β** **~ Norm (0, 100), μ ~ Norm (0,100), and σ^2^ ~ Unif (0,5).

We used R version 3.3.2 to package the data and WinBUGS 1.4.3 to fit the models. We ran three chains of 50,000 iterations each, with a burn‐in period of 10,000 iterations with no thinning. We assessed model convergence by visually examining the chains for mixing and using the Gelman and Rubin (1992) diagnostic (*R* < 1.1). We presented posterior means along with the 95% credible intervals (CrI) for all state variables of interest. We ran several versions of the multistate model to evaluate covariates, and potential lags in covariate effects, using deviance information criterion (DIC) for model selection (Appendix S1). Support for lagged effects was lacking in the dynamic model, so we fit only the full productivity model, without lagged effects.

## 3 RESULTS

**Figure 3:**
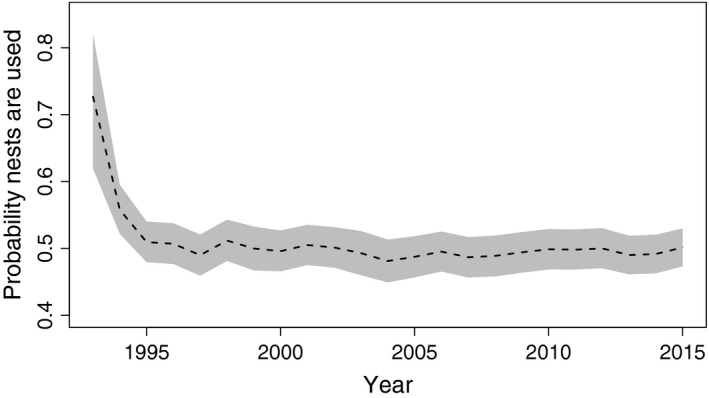
Mean posterior probability of bald eagle nest use in Lake Clark National Park and Preserve, Alaska, USA between 1993 and 2015. Nests are considered to be used when the occupied and successful states are combined. Use for the first year was fixed at the observed values, which were also the highest in 23 year time series

The dataset included 269 nests that were monitored at least once over the 23‐year period between 1993 and 2015. The constant model was most supported by our data, and we found little support for models containing covariates on the probability of transitioning among occupancy states. This means that nests were more likely to stay in the same state than transition to a new one and that our covariates failed to adequately explain variation in the transitions that did occur. Approximately half of the available nests had evidence of breeding activity (used) in any given year (0.51 CrI: 0.48–0.54), and this ratio remained constant, with no apparent trend during the 23 years of monitoring (Figure 3). Used nests had an even probability (0.56, CrI: 0.51–0.60) of succeeding. Interannual variation in these probabilities was low (Figure 3).

**Figure 4:**
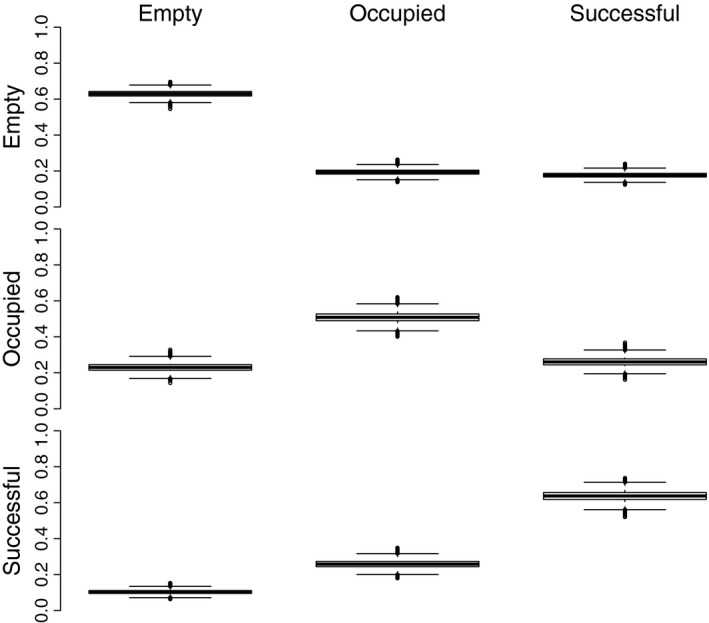
Posterior probability of each cell in the state and transition matrix for a 23‐year history of bi‐annual monitoring in Lake Clark National Park and Preserve, Alaska, USA. The diagonal cells represent the probability of staying in the same state, and the off‐diagonals represent the probability of transitioning from one state (*Y* axis) to another state (*X* axis). The boxplots depict the posterior distribution for each displayed transition parameter. The center line represents the median; the boxes represent the interquartile range (IQR); the whiskers represent the values that are within 1.5 * IQR, and the open circles represent values that are >1.5 * IQR

Unoccupied nests were more likely to remain unoccupied in the following year, than to transition to a higher occupancy state 0.63 (CrI: 0.59–0.66; Figure 4). Unoccupied nests were about equally as likely to transition to occupied 0.19 (CrI: 0.16–0.23) as successful 0.18 (CrI: 0.15–0.21; Figure 4). Successful nests were most likely to either remain in the successful state 0.64 (CrI: 0.58–0.69) or transition to the occupied state 0.23 (CrI: 0.22–0.30) between years, and had a low probability of becoming unoccupied 0.10 (CrI: 0.08–0.13; Figure 4). Occupied nests were equally likely to remain occupied 0.51 (CrI: 0.45–0.56) as to transition to another state in the following year, and transititions to either successful 0.26 (CrI: 0.19–0.28) or unoccupied 0.23 (CrI: 0.21–0.31) were about equally as likely (Figure 4).

**Figure 5:**
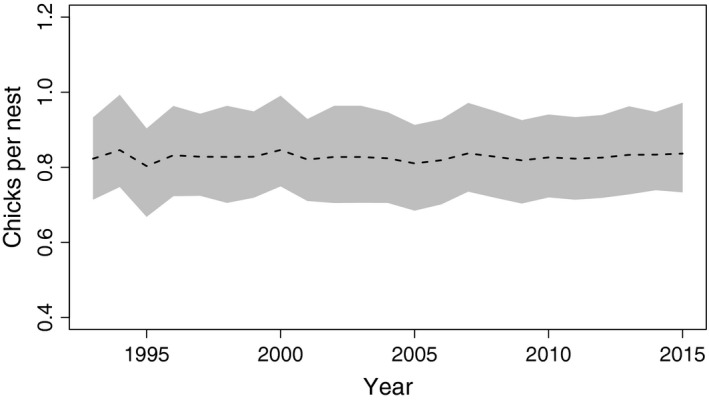
Temperature‐adjusted posterior mean (dashed line) and 95% credible intervals (gray shaded area) of the number of bald eagle chicks produced per used nest annually from 1992 to 2015 in Lake Clark National Park and Preserve, Alaska, USA

**Figure 6:**
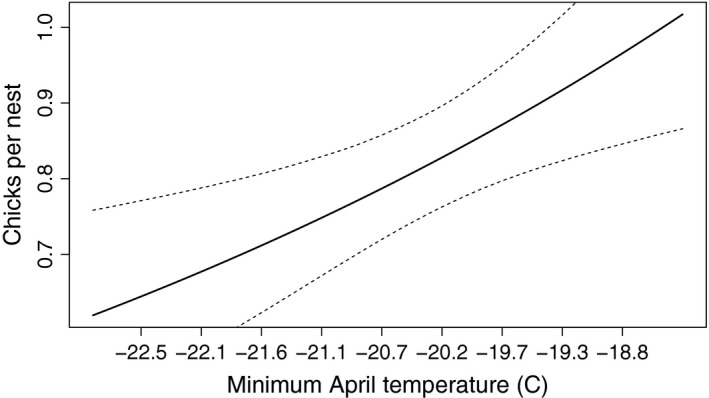
Predicted number of chicks produced per used bald eagle nest as a function of minimum April temperature in Lake Clark National Park and Preserve, Alaska, USA

Used nests (occupied and successful combined) produced 0.83 (CrI: 0.76–0.89) chicks, on average. We found no evidence of a trend in chick production over the 23 year period studied (Figure 5). Variation in the annual count of chicks produced per used nest was partially explained by minimum temperature recorded during the early nesting period (April); increasing April minimum temperature was associated with higher chick production (Figure 6). Other covariates (salmon escapement, and precipitation) were not supported.

## 4 DISCUSSION

Overall, our results were consistent with our predictions for a stable, naturally regulated bald eagle population in this largely undisturbed area in LACL. Our results suggest that high productivity and increasing trends observed in recovering populations (e.g., Grier, 1982; Watts et al., 2008) can be expected to stabilize at moderate levels (Smith et al., 2016). Overall, with the exception of uncertainty associated with correctly identifying occupancy state, we observed low variation in monitored metrics. This suggests that our methods provide reasonable sensitivity for detecting effects of new disturbances or stressors. We expect our findings will be useful both for assessing the impacts of any future large‐scale disturbances that might occur in LACL, as well as providing a useful reference for studies in other areas in North America, where human disturbance plays a larger role or where populations are still recovering.

Some bald eagle recovery plans mandate that chick productivity >1 chick per nest used for reproduction (‘occupied’ in Grier, Elder, Garamlich, Mathesen, & Mattsson, 1983), and this rate is often observed in expanding (Saalfeld, Conway, Maxey, Gregory, & Ortego, 2009; Smith et al., 2016; Watts et al., 2008), or dynamic (Anthony, Estes, Ricca, Miles, & Forsman, 2008) populations. Our observed estimate of chick productivity <1 per used nest, and stable occupancy dynamics are in line with populations elsewhere in Alaska (e.g., Steidl, Kozie, & Anthony, 1997; Zwiefelhofer, 2007) where density dependence may play a larger role (Elliott, Elliott, Wilson, Jones, & Stenerson, 2011). Our results strongly suggest that some mandated productivity guidelines may be unrealistic for evaluating successful recovery (Cruz et al., 2018), especially when there is consistent evidence that fecundity is density dependent in eagles (Elliott et al., 2011; Ferrer & Donazar, 1996; Mougeot et al., 2013).

Our observations of the proportion of used nests that were ultimately successful were similar to bald eagle populations elsewhere (Saalfeld et al., 2009; Smith et al., 2016). Stable probabilities of nest success are expected with healthy and recovering raptor populations where failure during the nestling stage is rare (Elliott, Moul, & Cheng, 1998). The propensity of nests to remain in the same state in subsequent years may indicate that territory quality or the quality of breeding pairs plays a role in determining occupancy state through time. Although not linked to an overall increase in reproductive output, the probability of nest success by raptors can be related to reuse (Beardsell, Gauthier, Therrien, & Bêty, 2016; Jiménez‐Franco et al., 2014). Furthermore, site fidelity by raptors has been related to previous nest success (León‐Ortega, Jiménez‐Franco, Martínez, & Calvo, 2017). We are unable to make conclusions regarding individuals because the population was unmarked; however, the relative importance of individual versus territory quality is an important question for future research.

Our finding that lower April minimum temperatures led to a decrease in chick production is consistent with past work showing that inclement weather depresses raptor reproduction (Beardsell et al., 2016; Gende, Willson, & Jacobsen, 1997; Steenhof et al., 1997). These results can also be interpreted as a positive reproductive response to spring warming, which could lead to increasing productivity in response to rising global temperatures (Fairhurst & Bechard, 2005). However, we found no evidence of warming‐related trends in occupancy dynamics or productivity in LACL.

We found no association between either the probability of nest success or productivity and variation in sockeye salmon escapement, despite studies showing that food availability was the most important factor affecting bald eagle reproduction (Dzus & Gerrard, 1993; Steidl et al., 1997). In Glacier Bay, Alaska most nests failed during incubation rather than when nestlings were present (Gende & Willson, 1997). We did not monitor nests intensively enough to determine the exact dates of nest failure, but if a similar pattern occurred in LACL, nest failure would occur before July when the sockeye salmon run begins in LACL (Young, 2014). Further, salmon are abundant in the spawning areas where they may be more available to eagles even later in the nesting season (Young, 2005), potentially making spawning sockeye more important for juvenile recruitment rather than chick production. Therefore salmon escapement may not be a very good measure of food limitation for bald eagle nest success or chick productivity in LACL, particularly if such limitation occurs well in advance of nesting as it does in golden eagles (Steenhof et al., 1997). Although salmon is an important component of the diet for bald eagles, their diet changes opportunistically throughout the season as fish availability changes (Armstrong, 2008), and forage fish such as herring (*Clupea pallasii*) may play a large role in pre‐nesting eagle diets (Gende et al., 1997). Bald eagles near Port Alsworth have been observed eating resident lake fish (e.g. lake trout—*Salvelinus namaycush;* least cisco—*Coregonus sardinella*), and waterfowl early in the nesting season (Mangipane personal observation). Therefore, a more targeted effort would be required to determine whether seasonal food availability affected nest success or chick production in LACL.

A caveat for interpreting our results is that we assumed that all states were observed perfectly. We know this assumption was likely violated based on a previous analysis of our field methods (Wilson, Phillips, & Mangipane, 2017); and imperfect detection very likely affected our estimates. For example, transitions from the occupied state were not different from what is expected from a random process and is most likely a result of imperfect separation of the unoccupied and occupied states. Imperfect state observation therefore most likely underestimated the probability of a nest occurring in either of the used (occupied and successful) states (Nichols, Hines, MacKenzie, Seamans, & Gutierrez, 2007). This should not result in biased estimates of trend, but may have impeded our ability to detect covariate effects (Gu & Swihart, 2004). Recent improvements in our bald eagle sampling protocol (Wilson, Weiss, et al., 2017) through the inclusion of a second survey in early spring, will allow us to fit a more robust dynamic occupancy model in the future. Additionally, many raptors have more than one nest in a territory (Millsap, Grubb, Murphy, Swem, & Watson, 2015). We were not able to distinguish between alternative nests and unused primary nests, and therefore urge caution when interpreting our results in terms of territories or territorial pairs.

Bald eagle nesting dynamics in LACL appeared to be stable for our 23‐year time series. Although this is the case, the observed relationship between temperature and productivity underscores the importance of continuing to monitor the species in Alaska where warming is expected to be most intense (IPCC 2013). In addition to temperature and precipitation effects, climate change and ocean acidification could change the base of the marine food web (Edwards & Richardson, 2004; Kroeker, Kordas, Crim, & Singh, 2010; Lam, Cheung, & Sumaila, 2016), the timing of salmon runs (Quinn, Hodgson, Flynn, Hilborn, & Rogers, 2007), and distribution of nesting substrate (Miller, Wilson, Sherriff, & Walton, 2017). These factors may influence bald eagle nesting populations in the future.

## CONFLICT OF INTEREST

None declared.

## AUTHOR CONTRIBUTIONS

Tammy L. Wilson conceived the original idea for the paper, organized the research team, formatted the data, coded the chick production model, wrote a majority of the content, and compiled author contributions into a complete manuscript. Dr. Wilson serves as the corresponding author. Joshua H. Schmidt provided project ideas and guidance, coded the dynamic multistate model, and contributed substantially to all stages of writing. Buck A. Mangipane provided project ideas and guidance, collected data for many years, compiled data, and contributed substantially to all stages of writing. Rebecca Kolstrom conducted the literature review and contributed substantially to all stages of writing. Krista Bartz compiled the salmon escapement data, provided expertise about salmon, and contributed substantially to all stages of writing.

## DATA ACCESSIBILITY

Data are free and available to the public through the National Park Service Integrated Resource Management Applications (IRMA) Data Store. https://irma.nps.gov/DataStore/Reference/Profile/2253441
