# Supplementary material for: Nest use dynamics of an undisturbed population of bald eagles
Source: Ecol Evol. 2018 Jun 27;8(15):7346–54. doi: 10.1002/ece3.4259 (PMC6106202; doi:10.1002/ece3.4259)
Supplement: Supplementary file 1 [file ECE3-8-7346-s001.docx]

This document contains supplementary materials for Nest use dynamics of an undisturbed population of bald eagles in Lake Clark National Park and Preserve, Alaska We present Summary plots of our data, and beta coefficient estimates for the multistate and generalized linear models.
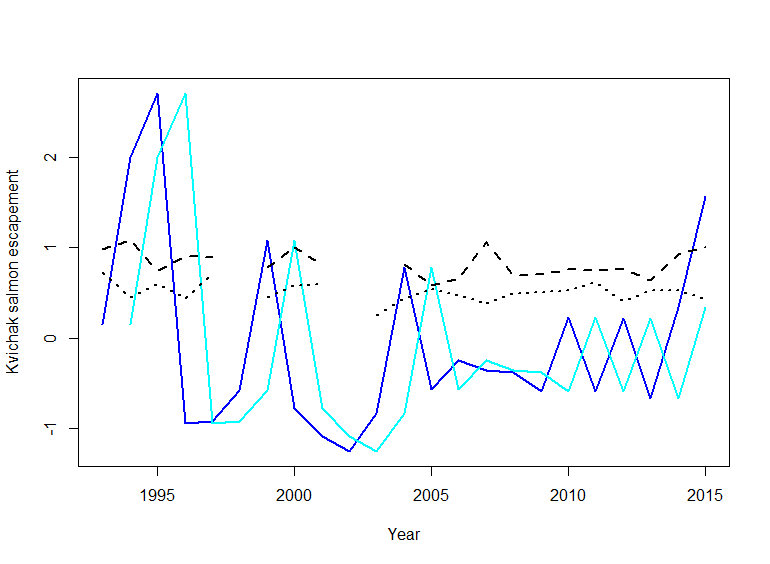


Fig. A.1. Summary plot of salmon escapement data from the Kvichak River in Alaska, USA. Salmon escapement data were centered and scaled by subtracting each value by the mean of all years and dividing by the standard deviation. The blue line represents escapement data for the current year, and the cyan line is shifted forward 1 year to evaluate lagged responses by nesting eagles. The dashed line represents the number of chicks produced per occupied nest, and the dotted line depicts the observed proportion of occupied nests.


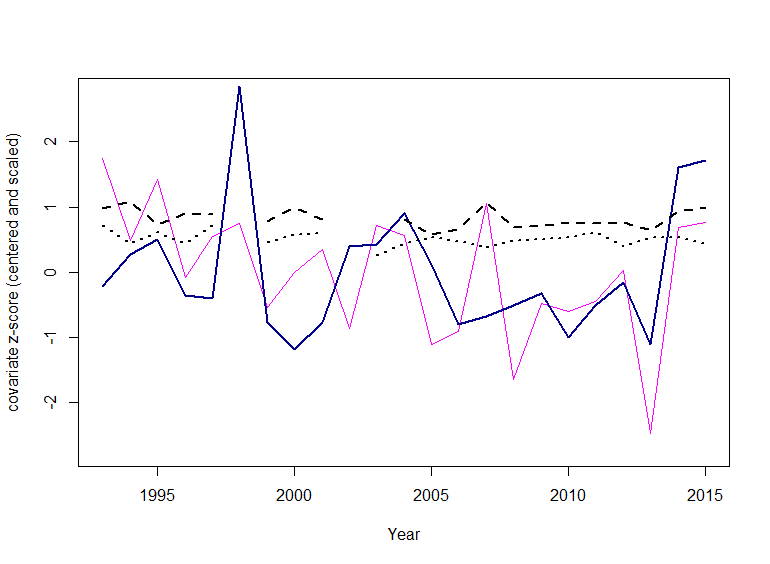


Fig. A.2. Summary plot of April minimum temperature, and May precipitation from the Bristol Bay, Alaska, USA climate division. Data were centered and scaled by subtracting each value by the mean of all years and dividing by the standard deviation. The dark blue line represents total precipitation in May of each year, and the magenta line is the minimum temperature in April. The dashed line represents the number of chicks produced per occupied nest, and the dotted line depicts the observed proportion of occupied nests.


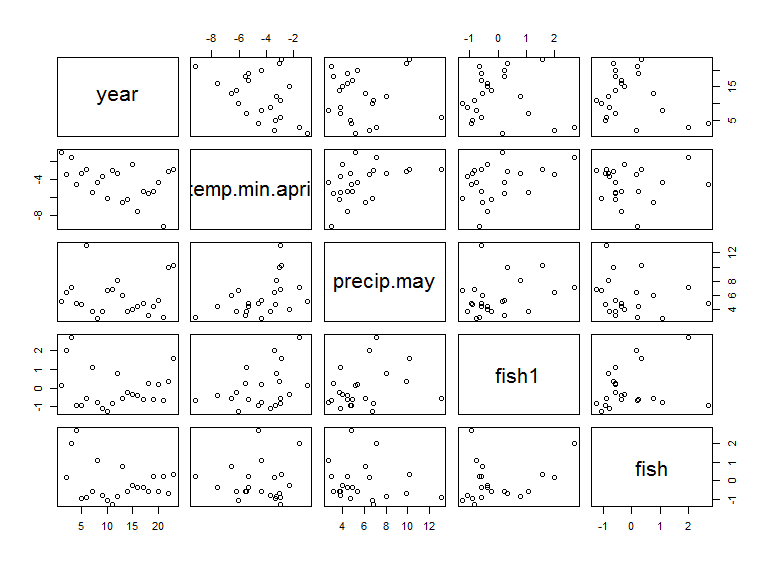


Pairs plot for all covariates used in the multistate and generalized linear models of bald eagle activity at nests in Lake Clark National Park and Preserve, Alaska, USA from 1993-2015. These plots are a visual indicator of collinearity. Year = year, temp.min.april = minimum temperature in April, precip.may = Total precipitation in May, fish1 = salmon escapment observed in that year, fish = salmon escapement in the previous year (lag). None of the covariates were correlated (Rpearson < 0.05)

Table A.1 Posterior means and 95% credible intervals (in parentheses) for beta coefficients from the multistate dynamic models of bald eagle activity at nests in Lake Clark National Park and Preserve (1993-2015). Coefficients describe the covariate effect on the probability of a nest staying in the same state (survival) or changing states (transition) from [the previous year] to [the observed year]. Mutually exclusive bald eagle nesting states are represented by the letters U, O, and S, where U is unoccupied, O is occupied, and S is successful. Bold text indicates parameter estimates where the 95% credible intervals do not overlap zero- an index of statistical significance.

| **Model** | **DIC** | **Delta DIC** | **Coefficients** | **Annual trend** | **Salmon escapement lagged by 1 year** | **Precipitation in May** | **Salmon Escapement** | **Minimum April temperature** |
| --- | --- | --- | --- | --- | --- | --- | --- | --- |
| Dot | 3528.439 | 0 |  |  |  |  |  |  |
| Trend | 3529.745 | 1.306 | B.survival[O] | -0.001  (-0.034,0.033) |  |  |  |  |
|  |  |  | B.survival[S] | -0.011  (-0.046,0.023) |  |  |  |  |
| Lagged salmon | 3530.516 | 2.077 | B.survival[O] |  | 0.072  (-0.165, 0.309) |  |  |  |
|  |  |  | B.survival[S] |  | -0.011  (-0.25, 0.232) |  |  |  |
|  |  |  | B.transition[U][O] |  | -0.071  (-0.251, 0.109) |  |  |  |
|  |  |  | B.transition[S][O] |  | 0.196  (-0.033, 0.434) |  |  |  |
|  |  |  | B.transition[U][S] |  | 0.017  (-0.282, 0.315) |  |  |  |
|  |  |  | B.transition[O][S] |  | -0.202  (-0.555, 0.153) |  |  |  |
| May Precipitation | 3532.498 | 4.059 | B.survival[O] |  |  | 0.023  (-0.249, 0.289) |  |  |
|  |  |  | B.survival[S] |  |  | 0.033  (-0.247, 0.32) |  |  |
|  |  |  | **B.transition[U][O]** |  |  | **-0.189**  **(-0.37, -0.008)** |  |  |
|  |  |  | B.transition[S][O] |  |  | 0.005  (-0.244, 0.27) |  |  |
|  |  |  | B.transition[U][S] |  |  | 0.08  (-0.221, 0.393) |  |  |
|  |  |  | B.transition[O][S] |  |  | 0.202  (-0.184, 0.595) |  |  |

| **Model** | **DIC** | **Delta DIC** | **Coefficients** | **Annual trend** | **Salmon escapement lagged by 1 year** | **Precipitation in May** | **Salmon Escapement** | **Minimum April temperature** |
| --- | --- | --- | --- | --- | --- | --- | --- | --- |
| Salmon | 3535.638 | 7.199 | B.survival[O] |  |  |  | 0.051  (-0.179, 0.284) |  |
|  |  |  | B.survival[S] |  |  |  | 0.112  (-0.11, 0.345) |  |
|  |  |  | B.transition[U][O] |  |  |  | -0.163  (-0.338, 0.007) |  |
|  |  |  | B.transition[S][O] |  |  |  | 0.018  (-0.182, 0.22) |  |
|  |  |  | B.transition[U][S] |  |  |  | 0.165  (-0.106, 0.449) |  |
|  |  |  | B.transition[O][S] |  |  |  | 0.057  (-0.253, 0.383) |  |
| April Temperature | 3537.184 | 8.745 | B.survival[O] |  |  |  |  | 0.032  (-0.209, 0.28) |
|  |  |  | B.survival[S] |  |  |  |  | 0.085  (-0.156, 0.323) |
|  |  |  | B.transition[U][O] |  |  |  |  | 0.028  (-0.129, 0.191) |
|  |  |  | B.transition[S][O] |  |  |  |  | -0.089  (-0.313, 0.131) |
|  |  |  | B.transition[U][S] |  |  |  |  | 0.154  (-0.129, 0.439) |
|  |  |  | B.transition[O][S] |  |  |  |  | 0.171  (-0.17, 0.526) |
| Full model without a lag | 3560.318 | 31.879 | B.survival[O] | 0.003  (-0.035, 0.042) |  | -0.014  (-0.387, 0.361) | 0.061  (-0.219, 0.344) | 0.012  (-0.308, 0.335) |
|  |  |  | B.survival[S] | -0.002  (-0.049, 0.045) |  | -0.079  (-0.532, 0.362) | 0.115  (-0.171, 0.403) | 0.063  (-0.313, 0.453) |
|  |  |  | B.transition[U][O] |  |  | -0.22  (-0.45, 0.01) | -0.143  (-0.346, 0.058) | 0.197  (0, 0.403) |
|  |  |  | B.transition[S][O] |  |  | 0.054  (-0.282, 0.395) | 0.053  (-0.199, 0.314) | -0.142  (-0.42, 0.129) |
|  |  |  | B.transition[U][S] |  |  | -0.081  (-0.483, 0.333) | 0.154  (-0.196, 0.511) | 0.134 (  -0.19, 0.473) |
|  |  |  | B.transition[O][S] |  |  | 0.207  (-0.29, 0.729) | -0.106  (-0.527, 0.305) | 0.142 (  -0.275, 0.575) |

Table A.2 Posterior means and 95% Credible intervals (in parentheses) for beta coefficients from the full generalized linear model of chick production at occupied nests in Lake Clark National Park and Preserve (1993-2015). Bold text indicates parameter estimates where the 95% credible intervals do not overlap zero- an index of statistical significance.

| **Model** | **Annual trend** | **Precipitation in May** | **Salmon Escapement** | **Minimum April temperature** |
| --- | --- | --- | --- | --- |
| Full | 0.003  (-0.014, 0.02) | -0.014  (-0.175, 0.147) | -0.009  (-0.115, 0.098) | **0.135**  **(0.013, 0.257)** |
